# Rapid profiling of triglycerides in human breast milk using liquid extraction surface analysis Fourier transform mass spectrometry reveals new very long chain fatty acids and differences within individuals

**DOI:** 10.1002/rcm.8465

**Published:** 2019-07-16

**Authors:** Albert Koulman, Samuel Furse, Mark Baumert, Gail Goldberg, Les Bluck

**Affiliations:** ^1^ Cambridge Lipidomics Biomarker Research Initiative, Elsie Widdowson Laboratory MRC HNR Cambridge CB1 9NL UK; ^2^ Lipid Profiling Signalling group, MRC HNR Cambridge CB1 9NL UK; ^3^ Core Metabolomics and Lipidomics Laboratory, MRL Institute of Metabolic Science Level 4, Pathology Building, Addenbrooke's Hospital Cambridge CB2 0QQ UK; ^4^ Department of Biochemistry University of Cambridge Hopkins Building, Tennis Court Road Cambridge CB2 1QW UK; ^5^ Advion Ltd Kao Hockham House, Edinburgh way Harlow Essex CM20 2NQ UK; ^6^ Nutrition and Bone Health Group, MRC HNR Cambridge CB1 9NL UK; ^7^ MRC Keneba The Gambia, Calcium, Vitamin D & Bone Health Group Banjul Gambia; ^8^ The Gambia Physiological Modelling of Metabolic Risk, MRC HNR Cambridge CB1 9NL UK

## Abstract

**Rationale:**

We describe a novel method for preparing milk samples and profiling their triglyceride (TG) fractions. This method was used to explore how the TG profile of milk modulates as lactation progresses and how the TG profile differs between breasts.

**Methods:**

Fresh milk was spotted onto Whatman filter paper and air‐dried. Liquid Extraction Surface Analysis coupled to Fourier Transform Mass Spectrometry (LESA‐MS) was adapted for molecular profiling. Collision‐Induced Dissociation (CID) was used to profile fatty acid residues.

**Results:**

LESA‐MS produced the relative abundances of all isobaric TGs described and showed that mammary glands within one individual can produce a different profile of TGs. CID was used to uncover the configuration of isobaric triglycerides, indicating the relative amounts of the fatty acids contributing to that triglyceride's mass. This also indicated the presence of very long chain fatty acids (C26:0 and C26:1) that have not been reported before in human breast milk.

**Conclusions:**

We conclude that spotting on paper and the use of LESA‐MS and CID on milk spots is not only a means for analysing milk in unprecedented detail for this preparation time, but is also amenable to conditions in which collecting and storing fresh milk samples for detailed profiling is prohibitively difficult.

## 1 INTRODUCTION

There is an increasing body of evidence that suggests that breast milk is important not only for the short‐term health and development of infants,^1^ but also for longer term health, cognition and reduction of risk of disease.^2^, ^3^, ^4^ To obtain a better understanding of how breast milk affects infants' health, as well as how the maternal diet and lifestyle affect breast milk composition, it is necessary to be able to measure the composition of human breast milk in detail, including its richest source of energy, triglycerides (TGs).

TGs are at least 50× as abundant as phospholipids in milk.^5^, ^6^ There are many studies on the total quantity of TGs in milk,^7^, ^8^, ^9^ but molecular profiling has thus far typically been restricted to gross profiles of the fatty acid (FA) composition. Thus, detailed information about the relative abundance of individual isoforms of TGs is lacking, as is an understanding of the kinetics of their production during human lactation. This paucity of data can be ascribed to a lack of specific and adequate methods for profiling TGs that are compatible with the need for running larger numbers of samples.

The gross FA profile of a biological sample typically involves preparation of Fatty Acid Methyl Esters (FAMEs) that are then profiled by gas chromatography (GC).^7^ This approach has a long history, including in studies of milk.^10^ This commonality facilitates the interpretation and comparison of results between studies. However, when the TG fraction is hydrolysed, information about the combinations of the acids in TGs and their positions on the glyceryl moiety is lost. Furthermore, the long sample preparation time required for this method invites variability and prohibits the profiling of larger sample numbers and thus bigger cohorts. Typical analysis times using this method are 30–60 min per sample.

Methods developed more recently have yet to gain significant traction in studies on milk. The analysis of TGs using high‐performance liquid chromatography (HPLC) is time consuming and lacks resolution.^11^ The combination of preparative HPLC or thin‐layer chromatography (TLC) to separate the triglycerides, hydrolysis, preparation and analysis of the methyl esters of constituent fatty acids by GC is detailed but only rarely performed^12^ as it too is time consuming and not really compatible with larger sample numbers. Very recently, liquid chromatography/tandem mass spectrometry (LC/MS^2^) has been used to profile the TG fraction of human milk.^13^, ^14^, ^15^, ^16^ However, this method suffers from poor fractionation of TGs by reversed‐phase chromatography and the solvents necessary for normal‐phase chromatography reduce the already poor ionisation efficiency of TGs.^17^

Paper chromatography was used for several decades because of its simplicity and effectiveness, but in recent years its use has declined due to lack of resolution and poor compatibility with sensitive detection methods. However, paper is an attractive medium for short‐term sample storage as it is amenable to anaerobic storage at low temperatures and does not require anti‐coagulants. Second, it requires only a small sample volume (10–50 μL or, in other words, just one drop) and little subsequent sample preparation.^18^ This contrasts with the collection of fresh milk samples that are required for LC/MS^2^ and other methods, and are typically considerably larger and more difficult to store than dried milk spots.

Luckily, the introduction of ambient ionisation methods such as Desorption Electrospray Ionisation (DESI) and Direct Analysis in Real Time (DART) have made it possible to revisit the use of paper for sample spotting and make it compatible with state‐of‐the‐art mass spectrometry. Furthermore, recent work with blood samples has shown that blood spots on filter paper can be used to obtain a representative lipid profile when compared with fresh blood or plasma.^19^, ^20^ In that work, it was necessary to make adjustments for the presence of artefacts (e.g. oxidised lipids),^19^ which may also appear in biological samples such as milk. However, sample preparation and running times were not excessive.

The need for convenient storage of milk samples and for processing larger numbers of samples, and the suggestion that paper may be a suitable medium, led us to the hypothesis is that filter paper is a suitable matrix for milk samples. Further, we suggest that it will absorb proteins and salts, the latter potentially interfering with lipid ionisation, leaving fat globules on the surface of the paper in a manner for analysis by ambient ionisation techniques such as Liquid Extraction Surface Analysis (LESA). We tested the hypothesis using a LESA coupled to a high‐resolution mass spectrometer (e.g. Fourier transform mass spectrometry (FTMS)), in order to obtain a detailed triglyceride profile of milk fat. Established multivariate data analyses were used to explore the data.^21^ Qualitative differences in isomeric TGs were determined using a linear ion trap.^22^

In this proof‐of‐principle research paper, we demonstrate how LESA‐FTMS can be used for the relative quantification of the TGs in breast milk. We developed the method and tested it on human breast milk from a small cohort of women from The Gambia, and compared the relative TG profiles with values reported already.

## 2 EXPERIMENTAL

### 2.1 Chemicals

Solvents of at least HPLC grade were purchased from Sigma‐Aldrich Ltd (Gillingham, Dorset, UK) and were not purified further. Lipid standards were purchased from Avanti Polar Lipids (Alabaster, AL, USA; *via* Instruchemie, Delfzijl, The Netherlands) and used without purification. Consumables were purchased from Sarstedt AG & Co. (Leicester, UK).

### 2.2 Lipid standards

In this study we used 0.6 μM 1,2‐di‐*O*‐octadecyl‐*sn*‐glycero‐3‐phosphocholine, 1.2 μM 1,2‐di‐*O*‐phytanyl‐*sn*‐glycero‐3‐phosphoethanolamine, 0.6 μM C8‐ceramide, 0.6 μM *N*‐heptadecanoyl‐D‐*erythro*‐sphingosylphosporylcholine, 6.2 μM undecanoic acid, and 0.6 μM trilaurin.

### 2.3 Ethics

All procedures were performed in accordance with the ethical standards of the institutional and/or national research committee and with the 1964 Helsinki declaration and its later amendments or comparable ethical standards. All mothers gave informed written consent after an oral explanation in the local language. The study was approved by the MRC/Gambian Government Ethics Committee.

### 2.4 Sample preparation

The milk was thawed, agitated and spotted on Whatman filter paper (Cat. No 1001 055) and air‐dried under a gentle flow of N_2_. Samples were used immediately.

### 2.5 Mass spectrometer

All samples were analysed using a Traversa NanoMate system (AdvionBioSciences, Inc., Ithaca, NY, USA) coupled to either an Exactive or an LTQ Velos Orbitrap mass spectrometer (both instruments from Thermo Scientific, Hemel Hempstead, UK). A nanoelectrospray ionization voltage of 1·55 kV and gas pressure of 0·2 psi was applied in all experiments. Customised robotic arm movements and custom liquid handling for a surface analysis were set up in the AUI panel of the ChipSoftManager software controlling the NanoMate. The Exactive was set up to collect data in positive mode with 1 Hz scan rate for maximum resolution. The LTQ Velos Orbitrap was set to collect data in positive mode with the following five scan events (SE):

SE1: FTMS 300–2000 *m/z* (res = 100000);
SE2: data‐dependent ITMS MS^2^ of most intense ion from parent list (fragmentation settings: Activation type = CID; normalized collision energy = 35%; isolation width = ±1 *m/z*; activation Q = 0·25; activation time = 30 ms; minimal intensity = 500);
SE3: data‐dependent IMTS MS^3^ of most intense ion from MS^2^ (same setting as SE2, except isolation width = ±2.5 *m/z*);
SE4: data‐dependent ITMS MS^3^ of 2nd most intense ion from MS^2^ (same setting as SE3);
SE5: data‐dependent IMTS MS^3^ of 3rd most intense ion from MS^2^ (same setting as SE3).

The Parent list comprised the ammonium adducts of all possible TG lipids (up to ca. 70 masses could be fragmented per sample analysis; see Table S1 (supporting information) for complete list).

### 2.6 Experimental plan

Mothers in rural areas of The Gambia typically introduce complementary foods after about 4 months and continue to breast feed for 18–24 months. The breast milk samples used in the analyses described in this paper were serendipitous collections from a longitudinal study of maternal calcium ion supplementation conducted in the villages of Keneba and Manduar in the West Kiang region of The Gambia.^23^ Only samples from women who were followed up were included in the final analyses. The samples were collected at 2, 13 and 52 weeks lactation from each woman and from each breast. Samples were frozen immediately after collection and stored at −20°C at MRC Keneba. They were shipped frozen to the UK on dry ice and stored at −80°C until analysis.

## 3 RESULTS

### 3.1 Spotting and sample preparation

Milk was spotted on paper with careful drying by gaseous nitrogen in order to obtain small and compact spots. This is possible by hand‐spotting, though reproducibility was better when using automated spotting or larger sample volumes. When the droplet of the extraction solvent did not make proper contact with the spot in the middle, or when the fat layer was compromised, triglycerides (TGs) appeared as only minor constituents and spectra were inconsistent. During development, we therefore spotted each sample nine times and discarded all analyses that showed poor extraction. Good spotting appears to be important for the success of this method as the quality of LESA is dependent on the formation of an intact hydrophobic layer of fat globules on the paper in combination with the extraction solvent making contact with the fat layer in the middle of the spot to prevent capillary forces of the paper draining the extraction solvent.

We tested a range of solvents for preparing dried milk spots on paper and found that methanol was optimum, with sufficient extraction efficiency but without the problem of the extraction solvent dispersing into the paper. We doped the methanol with ammonium acetate (20 μM) to provide enough ammonium to facilitate the ionisation of the (uncharged) TGs. The extraction efficiency of the lipids from the surface of the paper in LESA^24^ is dependent on the physicochemical properties of the surface as well as the properties of the liquid used for the extraction. The analytes need to diffuse rapidly into the extraction solvent, but the surface tension of the droplet of the extraction fluid should not be broken by the surface analysed, because that impairs an effective aspiration of the droplet after extraction.

### 3.2 Data acquisition

The full mass spectrum for each sample, averaged over 10 scans, was exported from the Xcalibur software into MS Excel and searched for the intensities of all the theoretical TGs (Table S1, supporting information). These peak areas were then averaged over three repeat analyses of the same sample in order to obtain the final value presented here.

### 3.3 Triglyceride profile from individuals

**Figure 1:**
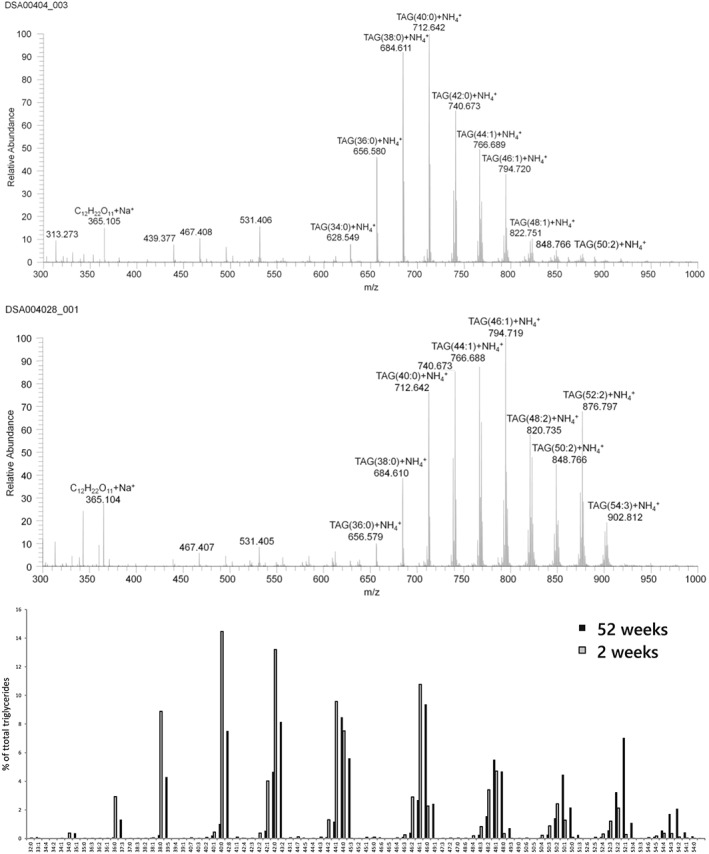
LESA‐MS^1^ spectrum of an on‐paper spotted sample of human breast milk collected at 2 weeks (top) and 52 weeks (middle) of lactation from one individual. Bottom bar graph shows the levels of each triglyceride signal as a percentage of the total triglyceride signal

Mass spectra obtained from milk samples prepared and analysed in this way (spotting/LESA‐MS) are shown in Figure 1. The samples from each individual comprise milk collected from both breasts. Samples were profiled within about 48 h; storage of milk spots for prolonged periods (>14 days) at room temperature led to sample degradation. The latter is ascribed to oxidation of the olefin bonds. Additionally, we show a subsection of the mass spectrum of a sample spotted on the same day and either profiled immediately or after storage for 14 days at room temperature in the dark. There was a clear increase in the abundance of oxidised TGs in samples analysed after 2 weeks of storage at room temperature and in partially aerobic conditions. However, there was no measurable effect within 48 h. The extent of oxidation was limited to 2–3% of the original peak and without any distinct effect on the overall TG profile. Oxidation could be slowed down considerably by storing the dried milk spots in a freezer and under an inert atmosphere.

### 3.4 Triglyceride profile through time

**Figure 2:**
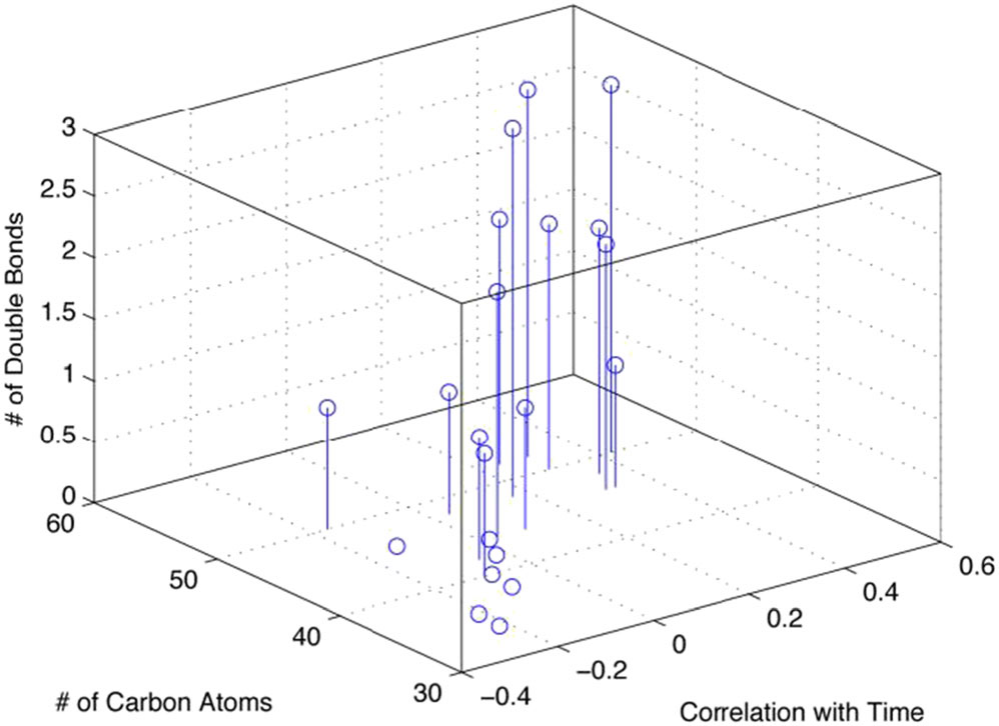
Correlation (x‐axes) between stage of lactation (2 and 50 weeks post partum) and number of double (y‐axes) bonds and number of carbons (z‐axes) [Color figure can be viewed at wileyonlinelibrary.com]

In order to determine the global changes in lipid profile over 50 weeks of lactation, the data from the two breasts were averaged and used in a multivariate analysis to determine underlying patterns of correlation (see Figure 2). For this approach, only data points that showed high reproducibility across all samples were used. The multi‐variate data analysis revealed clear positive correlations between the number of carbons and number of double bonds, and number of weeks of lactation.

### 3.5 Identifying isobaric TGs

**Figure 3:**
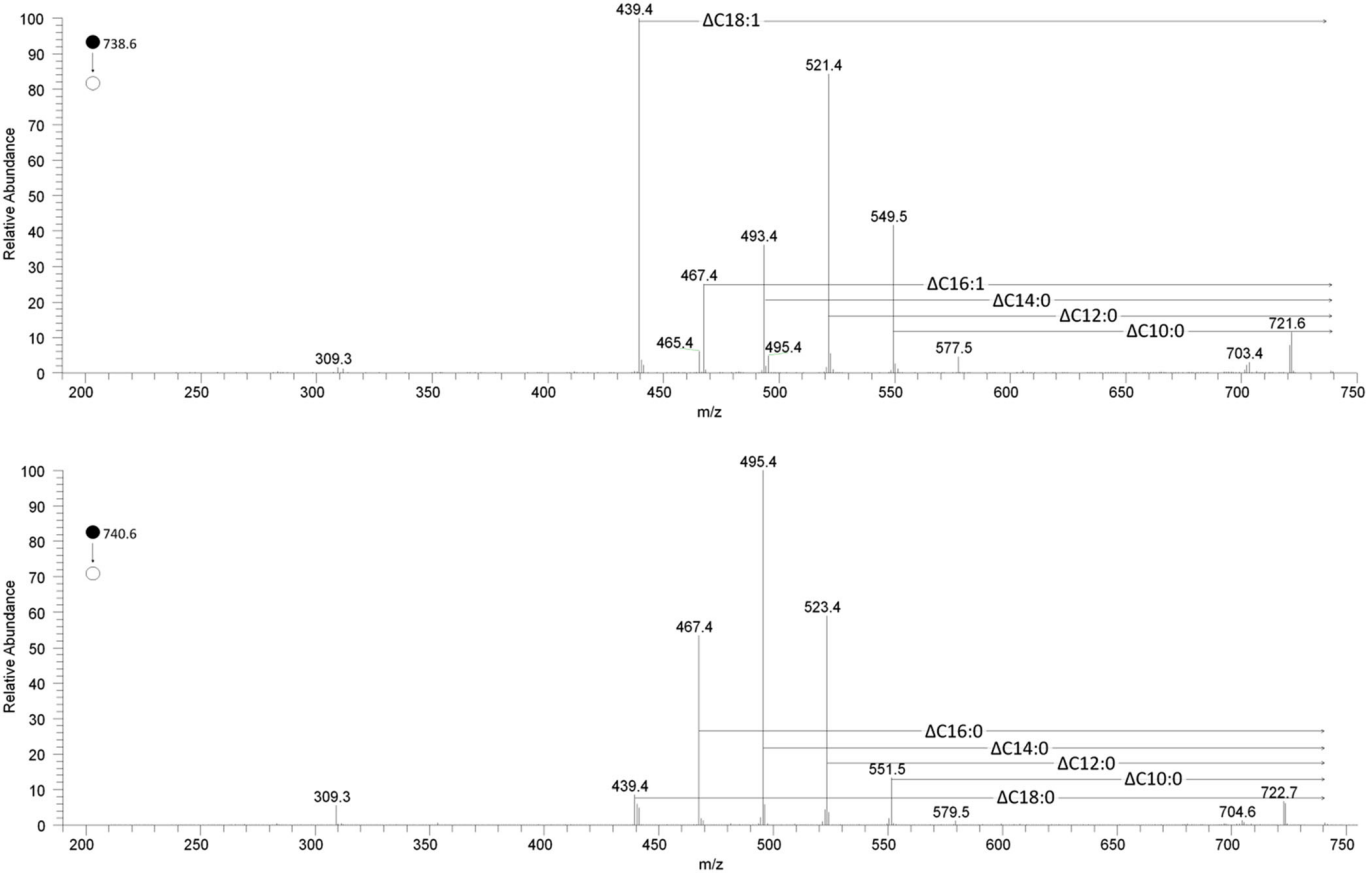
MS^2^ spectra of the ammoniated ions of TG(42:1) (top trace) and TG(42:0) (bottom trace) [Color figure can be viewed at wileyonlinelibrary.com]

A number of isoforms of TGs are difficult to identify as they are isobaric to others, e.g. the ammonium adduct of TG(36:2) will give a peak at 652.551 *m/z* but the configuration could be 18:1/14:1/4:0, 18:2/14:0/4:0, 18:2/12:0/6:0, amongst others. The intensity of this peak is therefore the sum of the intensity of ions that all have the same molecular formula (i.e. the sum of all the molecular configurations of that isoform). Collision‐induced dissociation (CID) was used to identify the configuration of isobaric TGs, revealing the identities of the three fatty acid residues (FARs). MS/MS spectra of fragmentation to diglycerides of ammoniated ions of TG(42:0) and TG(42:1) are shown in Figure 3. The loss of FARs from the TGs is shown, providing an insight into the relative distribution of the fatty acids in that particular group of isobaric TGs. For TG(42:0) the most abundant loss was C14:0, while for TG(42:1) it was C18:1. This suggests that unsaturation is chiefly invested in the C18:1 FAR and only about 30% is C16:1, with less than 10% being C14:1. The presence of C18:1 correlates with that of shorter chain fatty acids such as C10:0, whose abundance was at least twice as high in TG(42:1) than in TG(42:0). Unfortunately, this method is similar to other mass spectrometry approaches in that it is not possible to identify the position of FARs on the glyceryl moiety (*sn‐*1, ‐3 or ‐2), or the position or geometry of the double bonds.

### 3.6 Very long chain fatty acids

**Figure 4:**
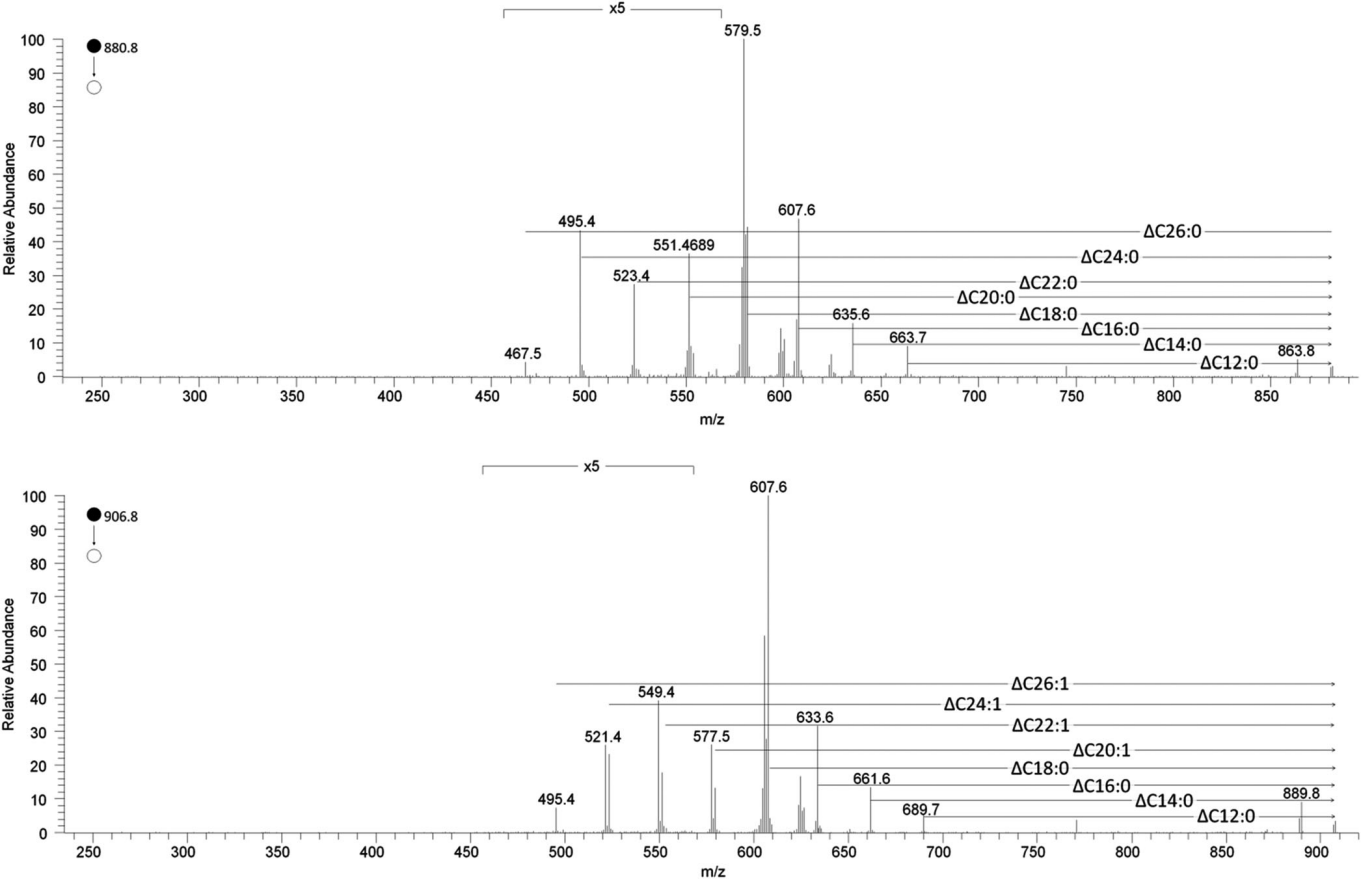
The MS^2^ spectra of TG(52:0) (top graph) and TG(54:1) showing the relative abundance of the different fatty acids contributing to these triglycerides. This has not previously been reported in human milk samples

Despite the limits of MS in determining the regiochemistry of FARs in TGs, the results obtained using this approach have provided novel insights. As well as evidence for short‐ and medium‐length carbon chains (around C10:0 and C16–18, respectively), samples profiled using LESA‐MS also indicate the presence of longer FARs. Our results are consistent with the several reports of C24:0 and C24:1 FARs in the TGs of human milk,^25^, ^26^, ^27^ but the MS^2^ spectra of TG(52:0) and TG(54:0) in samples taken 14 days *post partum* showed losses corresponding to C26:0 FAs. Similarly, TG(52:1) and TG(54:1) showed losses corresponding to C26:1 (Figure 4). The presence of FAs with such long carbon chains has been reported in bovine milk,^28^, ^29^ but we could not find any reports in the literature of C26:0 and C26:1 in human milk. This result hints that VLFARs may be a feature of milk from all mammals and raises the question of their role *in vivo*.

### 3.7 Variation in the TG profile within individuals

**Figure 5:**
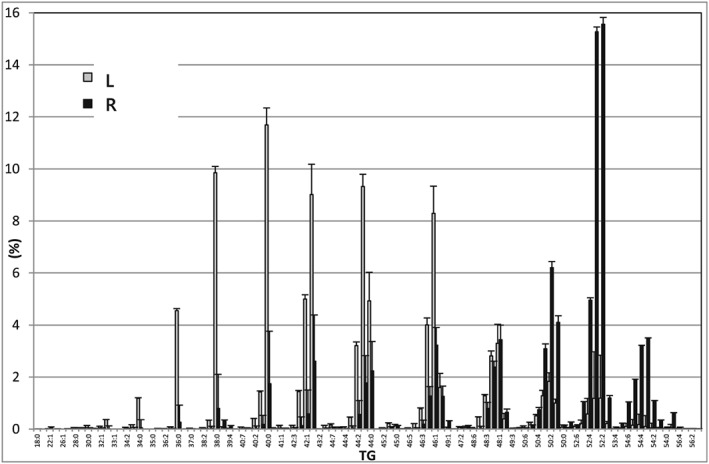
The average relative values of triglycerides in human breast milk collected from the two breasts at 13 weeks of lactation in one subject: Left (grey bars) and right (black bars). n = 4 measurements

We also used this pilot study to follow up published studies that indicate that provision of milk differs between breasts. Differences between breasts during the same lactation have been reported for volume of milk produced^30^, ^31^ and amount of fat;^32^, ^33^ however, the TG profile has not yet been reported. LESA‐MS was used to profile the TG fraction in milk from two different breasts during the same lactation and the results are shown in Figure 5.

In this example, the difference between breasts was clear for all aspects of the TG profile. One breast gave a profile that was high in lower molecular weight TGs while the milk of the other contained a higher level of TGs with a higher molecular weight. For example, the MS^2^ spectrum of TG(42:1) in the milk from one breast shows a greater loss of C12:0 (Figure S1s, supporting information), but a lower loss of C16:1, than in the other breast. There are also other, subtler differences. Spectra of TG(42:0) indicate that the abundance of TG(18:1/12:0/12:0) is more common than TG(18:1/14:0/10:0) in one breast, while the ratio is more similar in the other.

Further work is required to understand why different mammary glands in the same woman should produce milk with different TG profiles, or how this is controlled. However, spotting‐LESA‐MS may be amenable to research in this area. The spectra consist mainly of ammonium adducts of TGs, but diglycerides (as ammoniated ions), cholesterol lipids (as ammoniated ions) and phospholipids (as protonated ions) are also present. Furthermore, the results obtained by the LESA‐MS method are consistent with reports that the relative abundance of phospholipids is ~2%.^7^, ^27^

## 4 DISCUSSION

This study was undertaken in order to develop a method to profile milk that had been dried onto paper. The latter was intended as a means for investigating milk production in greater detail, for example in handling larger numbers of samples and from regions where collecting and storing large numbers of samples of fresh milk is impractical. The profiling results suggest that it is possible to carry milk samples on paper. The latter is reliant upon a consistent spotting technique and does not extend the shelf life of the samples at room temperature, however.

Molecular profiling of the samples from a small cohort from The Gambia is consistent with most of our current understanding of fat production in human breast milk. A well‐discussed problem in milk analysis is the homogeneity of the sample and how representative this sample is. The changes in composition of milk from a high concentration to a more aqueous composition during a single feed have been observed several times.^5^, ^34^ Similarly, significant differences in the milk composition produced by the two breasts at the same stage of lactation have been reported.^30^, ^31^ Purifying these changes in detail demands great care with sample collection, storing and aliquoting. Understanding the dynamics of the lipid composition and biological factors that drive these has been difficult to study as they demand fast sampling rates and analytical methods with high‐throughput capabilities. The LESA‐MS method described in this paper opens up the possibility of studying fat production in much more detail because of its low sample volume requirement and fast and inexpensive nature.

However, in order to broaden its use for studying populations, or even larger numbers of individuals, it may be useful to validate the method further. The major difficulty in developing the LESA‐MS method for milk TGs was the validation. The experimental barrier exists because it is difficult to impregnate an extant oil‐in‐water emulsion with internal standards. The introduction of organic solvents such as hexane or dichloromethane disrupts the hydrophobic effect that drives their formation in water, and production of synthetic oil bodies comprising lipid and triglyceride standards has yet to be realised.

Our review of the literature shows that this problem has been largely avoided in the validation of other methods. In most reports the internal standards were added after extraction of milk or in the extraction solvent,^35^ or no internal standard was used.^6^, ^26^, ^36^ This is a valid approach because the data can be compared with the direct analysis of standards. We therefore limited validation to demonstrating repeatability of the method using quality control samples. This approach is limited in that it correlates closely with the concentration of the TGs, with less abundant TGs proving less repeatable. Across the 30 samples we found that we could measure 23 TGs with a coefficient of variance of <20%, regarded here as acceptable. The effect of this limit can mean that changes in concentration of TGs can be difficult to plot accurately. For example, the abundance of TG(54:3) may be around 0·01% 2 weeks *post partum*, but is about 3% at week 52. Without more thorough validation it is not possible to say whether it is produced from the outset or whether production begins in the first month *post partum*. However, such large relative changes in TGs have not been reported in so much detail yet, and so there is scope to build on these results and those of other formative studies.^37^

As well as changes over time, there is a consistent difference between the milk produced in the two breasts at the same feed in the individual sample donors of the present study. Evidence from this study shows that one breast yields milk with a profile of higher shorter‐chain (~C14) saturated fatty acids while the other breast shows a higher abundance of lipids with longer (~C18) unsaturated fatty acids. Also, the fragmentation data of the TGs shows differences in the composition of isobaric TGs.

This is perhaps surprising because the biosynthesis of FAs for milk fat production in the breast is generally in the range of 10–14 carbons per chain.^38^ The breast that produces milk with a higher concentration of saturated shorter‐chain fatty acids might be using higher amounts of sugars for *de novo* synthesis of FAs, whilst the other breast is more like a conduit for FAs from the circulation.

Another indication of the systemic import of FAs from the circulation is the qualitative details obtained by CID of the presence of very long saturated and mono‐unsaturated FAs with C26:0 and C26:1 configurations. This is the first report on the presence of such very long chain FAs in human breast milk, as previous work reported up the presence of FAs up to C24. There was evidence for C26 FAs at all three time points, though this appears to be clearer in the earlier samples (2 and 13 weeks). It is not clear why this should be; however, as the abundance of a number of FAs changes through lactation, further work is required.

Further work is also required to understand the timing of the supply and the role of very long chain fatty acids supplied in breast milk. A number of studies show that C20 and C22 FAs have a role in neural development,^39^ cognition^40^, ^41^ and behaviour.^42^ The role(s) of C24 and C24:1 and even C26 in humans has received very limited attention and it is not clear if C24 and C26 are essential FAs.^43^ These very long chain fatty acids are used in sphingolipid biosynthesis, which are essential in epidermal keratinocytes and male germ cells, along with much longer fatty acids (up to C36).^44^, ^45^ Humans are unable to make docosahexaenoic acid (DHA, C22:6_ω‐3_) because we do not possess the appropriate dehydratases to biosynthesise C22:6 from C24:6.^46^ What is known is that longer FARs, especially ones that are saturated or only singly unsaturated, form more ordered lipid aggregations,^47^, ^48^ which is consistent with the lateral inhomogeneity of liquid ordered regions and lipid rafts. The presence of such lipids therefore limits membrane fluidity.

It is important to emphasise that this is a proof‐of‐principle investigation based only on five individuals. These cannot be regarded as representative of Gambian or other women in general and these findings cannot be extrapolated. It is therefore not clear if these very long fatty acids in TGs are a common phenomenon in breast milk or if this is particular for these five individuals. However, as other studies that have collected milk samples from either mammary gland have shown that milk production varies between them,^30^, ^31^ the distinction between the TG profiles of milk from different breasts during the same lactation may be more typical.

Nevertheless, more studies are required, comprising larger numbers of subjects from different populations, at different stages of lactation and different stages of a feed to further our understanding of milk production in humans.

## Supporting information

Figure S1: The 800–900 m/z section of the spectrum obtained by LESA of a dried paper milk spot sample, either within 2 hours of spotting (top trace) or after 2 weeks (bottom trace). The bottom trace shows oxidation of the unsaturated fatty acids.

Table S1. The search list of triglycerides used for automated identification of m/z signals. ‘Carbons’, refers to the number of carbons in the fatty acid residues of the TAG.

Supporting info item
