# Supplementary material for: Rapid profiling of triglycerides in human breast milk using liquid extraction surface analysis Fourier transform mass spectrometry reveals new very long chain fatty acids and differences within individuals
Source: Rapid Commun Mass Spectrom. 2019 Jul 16;33(15):1267–76. doi: 10.1002/rcm.8465 (PMC6772081; doi:10.1002/rcm.8465)
Supplement: Supplementary file 1 — Figure S1: The 800–900 m/z section of the spectrum obtained by LESA of a dried paper milk spot sample, either within 2 hours of spotting (top trace) or after 2 weeks (bottom trace). The bottom trace shows oxidation of the unsaturated fatty acids. [file RCM-33-1267-s001.docx]

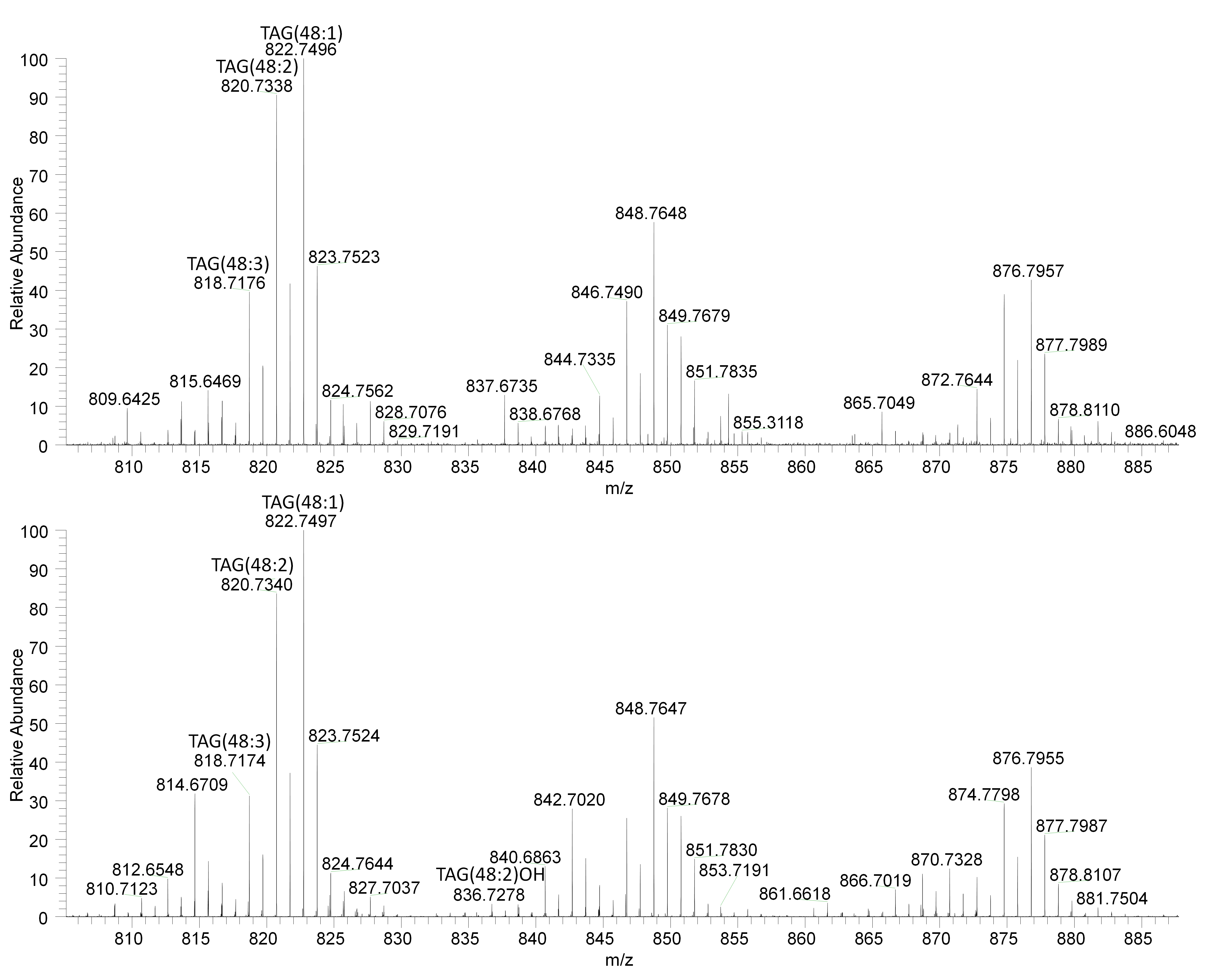


Figure 1s: The 800-900 *m/z* section of the spectrum obtained by LESA of a dried paper milk spot sample, either within 2 hours of spotting (top trace) or after 2 weeks (bottom trace). The bottom trace shows oxidation of the unsaturated fatty acids.
